# Neurochemical characteristics of pathological tissues in epilepsy: A preliminary ^1^H MR spectroscopy study at 7 T

**DOI:** 10.1016/j.ejro.2025.100640

**Published:** 2025-02-27

**Authors:** Lijing Xin, Philippe Reymond, José Boto, Frederic Grouiller, Serge Vulliemoz, Francois Lazeyras, Maria Isabel Vargas

**Affiliations:** a Center for Biomedical Imaging, Ecole Polytechnique Fédérale de Lausanne, Lausanne, Switzerland; b Division of Neuroradiology, Geneva University Hospitals and University of Geneva, Switzerland; c Swiss Center of Affective Sciences, University of Geneva, Switzerland; d Center for Biomedical Imaging of Geneva and University of Geneva, Switzerland; e Division of Neurology, Neurosciences Department of Geneva University Hospitals and University of Geneva, Switzerland; f Clinique des Grangettes-Hirslanden and University of Geneva, Switzerland

**Keywords:** Epilepsy, MR spectroscopy, Non lesional, 7 T, Cortical dysplasia

## Abstract

**Background and purpose:**

This study aims to evaluate the neurochemical characteristics of pathological tissues by ^1^H magnetic resonance spectroscopy (MRS) in patient with pharmaco-resistant focal epilepsy at 7 T.

**Methods:**

Thirteen patients with drug-resistant epilepsy and focal seizure successfully underwent MRS examinations at 7 T MRI scanners. ^1^H MR spectra were acquired from two voxels (lesion side and contralateral side) using the semi-adiabatic spin-echo full-intensity localized spectroscopy (sSPECIAL) sequence. Metabolite levels were quantified from LCModel and reported as to total creatine ratio.

**Results:**

In comparison to the contralateral side, lesions in focal cortical dysplasia demonstrated significantly reduced macromolecule and N-acetyl aspartate, significantly increased total choline and glycine + myo-inositol, and a distinct reduction trend of glutamate.

**Conclusions:**

We conclude that performing MRS at high magnetic field offered the potential to reveal metabolic alterations in epilepsy lesions that may help to further understand the underlying pathophysiology of the disease.

## 1 Introduction

Epilepsy is a frequent neurological disorder affecting both children and adults that is characterized by a variety of symptoms, many of which can lead to death. Around 30 % seizures are not controlled by anti-epileptic drugs. However, when a focal epileptic activity can be identified using a thorough multimodal imaging work-up, the focus may be removed by surgery leading to seizure freedom or to a significant improvement.

Ultra-high field MRI offers higher signal-to-noise ratio, and spatial resolution relative to low to intermediate clinical magnetic fields (3 T and below). The application of MRI studies at 7 T have demonstrated promising results for the detection of inconspicuous epileptogenic lesions for a great number of MRI-negative patients at 3 T [1], [2], [3], [4].

^1^H magnetic resonance spectroscopy (MRS) allows non-invasive investigation of metabolic changes in different physiological and pathological conditions. MRS studies in epilepsy have revealed abnormal N-acetyl-aspartate (NAA), creatine (Cr) and choline (Cho) levels, for example in temporal lobe epilepsy [5], [6] and in drug-resistant epilepsy with focal cortical dysplasia [7]. It also shows the potential to predict temporal lobe epilepsy [8] and surgical outcomes [9].

So far, MRS studies of epilepsy have been largely performed at 3 T. Although high magnetic field strengths have shown great potential for improving the detection of metabolites, research studies in epilepsy at 7 T are sparse [4], [9]. The metabolites of studies performed at field strengths of 3 T or below often include those that occur more abundantly such as NAA, creatine, and choline. Enhanced spectral resolution and sensitivity at 7 T combined with advanced MRS methodologies [10] allows for neurochemical profiling including improved detection of glutamate and glutamine [11], the major inhibitory neurotransmitter (GABA) and the antioxidant (glutathione). In particular, glutamate-glutamine-GABA cycling dysfunction seems to play a central role in epilepsy [12]. Therefore, the aim of this study is to evaluate the extended neurochemical characteristics (including glutamate, glutamine, GABA and glutathione) of pathological tissues by ^1^H MRS in patients with drug-resistant epilepsy at 7 T.

## 2 Methods

### 2.1 Patients

Patients with drug-resistant epilepsy and focal seizure followed-up in our institution, most of them not presenting a clearly visible lesion on 3 T MRI were included in this study. The exclusion criterion was a contraindication to 3 T MRI examination. The study was approved by the ethics committee of the institution (no. 13–262). Patients participating in the study were given detailed information and signed a written informed consent. The study period was from July 2014 through January 2020, and 39 patients were included.

We performed cross validation with complementary information (clinical examination, EEG, PET) to identify the optimal voxel localization. Patients for whom this lesion was located at the fronto-basal and temporal pole regions or near the petrous bone were excluded due to magnetic field inhomogeneity and magnetic susceptibility artifacts.

We performed MR spectroscopy examinations in 17 patients. Some spectroscopy examinations were not retained due to an incomplete MRS scan of the contralateral region (n = 1), insufficient spectral quality related to patient movement (n = 1) and insufficient spectral quality (n = 2). Thirteen patients were thus included (54 % males) with a median age of 22 years, ranging from 14 to 48 years (demographic data in Table 1). Among them, three patients had focal cortical dysplasia, two patients had ganglioglioma, one with rare ectopic neurons of the subcortical white matter proved by histopathology, one with suspicion of dysplasia who is followed by MRI, and six patients had no visible abnormalities on morphological images, for whom putative locations were assessed by convergence of clinical and other imaging modalities (EEG, PET, SPECT).

**Table 1:** Demographic data for patients who underwent MRS scans (n = 13) including clinical presentation, seizure frequency, MR diagnosis and post-operative clinical outcome.

N°	Age (years) and Gender	Age at onset (years)	Clinical presentation	Seizure frequency /day	MR Diagnosis and/or proved diagnosis by histopathology	Post-operative clinical outcome
1	31 M	0.3	Partial complex seizures, secondary generalized	0.06	Suspicion of dysplastic area left temporo-occipital	No surgery
2	19 M	12	Focal frontal seizure at night	0.3–0.4	FCD type IIa	Surgery
3	19 M	18	Absence	2–3 episodes in total	Ganglioglioma	Focal and secondary generalized seizures
4	32 M	17	Simple sensitive partial seizure	2–3	Ganglioglioma	Seizure free
5	18 F	11	Partial complex seizures secondary generalized	1–2	FCD type IIa	Seizure free
6	29 F	10	Hemifacial and right hemi-bodies clonic, frequently secondary generalized	Frequent at the beginning. 5–6 seizures in total when adult	Rare ectopic neurons of the subcortical white matter	Seizure free
7	22 M	6	Late: nocturnal seizures once a month on waking with right leg hypoesthesia and convulsion paresis then generalized tonic-clonic movement	0.03	FCD (gamma knife)	Seizure free
8	21 M	10	Late: rare tonic-clonic bilateral seizures during sleep or alcohol consumption weaning	Some seizures /day	No visible lesion on the morphological imaging	No surgery
9	33 F	27	Focal left hemisphere. Few secondary generalized	Decreased from 5 to 6–1 under Lamotrigine	No visible lesion on the morphological imaging	No surgery
10	43 F	10	Atonic/astatic seizure with altered consciousness and bilateral tonic-clonic seizure	Some seizures /day^b^	No visible lesion on the morphological imaging	No surgery
11	48 F	22	Secondary generalized	0.33–0.4	No visible lesion on the morphological imaging	No surgery
12	14 M	12	Tonic or tonic-clonic allure	0.06–0.1^a^	No visible lesion on the morphological imaging	No surgery
13	19 F	6	Stable nocturnal frontal epilepsy type	One seizure in total in June 2019 under Carbamazepine, Brivaracetam, Zonisamide	No visible lesion on the morphological imaging	No surgery

FCD: Focal Cortical Dysplasia

a last seizure 4 days before 7T-MRI

b Last seizure reported in January 2018 under Topiramate

### 2.2 Magnetic resonance spectroscopy

All MR experiments were conducted on a 7 T/68 cm MR scanner (Siemens Healthcare, Erlangen, Germany) with a ^1^H quadrature surface coil or a single-channel quadrature transmit and 32-channel receive coil (Nova Medical Inc., MA, USA) depending on the location of the lesion. The dielectric pad [13] filled with a solution of deuterated water and barium titanate was used to enhance the local transmit field. Four imaging sequences were applied including 3D MP2RAGE, susceptibility-weighted imaging, diffusion tensor imaging and fluid-attenuated inversion recovery [3].

**Fig. 1:**
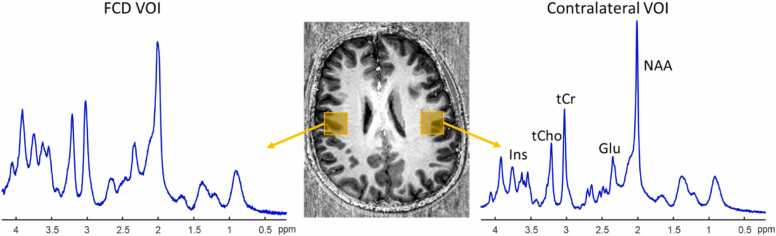
MRS voxel positions and ^1^H MR spectra from the control and lesion side of patient #2 with focal cortical dysplasia. Glu: glutamate, NAA: N-acetyl aspartate, tCr: creatine+phosphocreatine, tCho, total choline, Ins: myo-inositol.

T_1_-weighted morphological scan was obtained by the MP2RAGE sequence [14] (TR = 6000 ms, TE =3.1ms, TI1/TI2 = 800/2700ms, α1/α2 = 5^°^/7^°^, FOV = 210 × 210 × 160 mm^3^, matrix size = 320 × 320 × 256, bandwidth = 300 Hz/Px) for the positioning of the MRS voxels. B_0_ field inhomogeneities were minimized with first and second-order shims using FASTMAP [15]. For each patient, MR spectra were acquired from two voxels (lesion and contralateral side) using a semi-adiabatic spin-echo full-intensity localized spectroscopy (sSPECIAL) sequence [16], [17] with identical experimental parameters (TE = 12/16ms, TR = 6.2–8.0 s, VOI = 2–11 mL, averages = 32–120, bandwidth = 6000 Hz, 2048 no. of point) adapted for the coil used and location of the voxel (Fig. 1). For patients who did not demonstrate an apparent MR detectable lesion, the ‘lesion side’ voxel was positioned based on metabolic (PET-CT and SPECT), electrical (EEG) or clinical information. Outer volume suppression (OVS) with six saturation bands, and water suppression using variable power radiofrequency pulses with optimized relaxation delays (VAPOR) were performed prior to the localization.

Frequency drift and phase correction were applied prior to summation of multi-block spectra. Averaged MR spectra were analyzed by LCModel [18] (Stephen Provencher, Inc., Oakville, ON, Canada) with a basis set including 21 simulated metabolites spectra (alanine, ascorbate, aspartate, creatine, glucose, glycerolphosphocholine, glycine, glutamate (Glu), glutamine (Gln), glutathione, lactate, myo-inositol, scyllo-inositol, N-acetylaspartate (NAA), N-acetylaspartylglutamate, phosphocholine, phosphocreatine, phosphoethanolamine, serine, taurine, and γ-aminobutyric acid) and the experimentally measured macromolecular baseline. The analysis range was from 0.2 to 4.2 ppm. Metabolite levels with Crámer-Rao lower bound (CRLB) below 50 % were reported as a ratio of total creatine. All metabolite levels were calculated as a ratio of total creatine and relative metabolite changes between lesion and contralateral side were then calculated and reported as a percentage. Two-tailed paired *t*-test was used to compare neurochemical changes between the lesion and contralateral side.

## 3 Results

Thirteen patients were diagnosed on the basis of morphological MRI images. Among these patients, those whose diagnosis was confirmed by histopathology showed the following results: focal cortical dysplasia (FCD, n = 4, one with suspicion), ganglioglioma (n = 2), rare ectopic neurons of the subcortical white matter (n = 1). The remaining 6 cases had no MR visible lesion at 3 T and 7 T, However the clinical workout, EEG and PET/SPECT images pointed out a location most likely matching the epileptic focus. Due to the small sample size in certain groups, statistical analysis was performed in FCD and non-lesion patient groups.

**Fig. 2:**
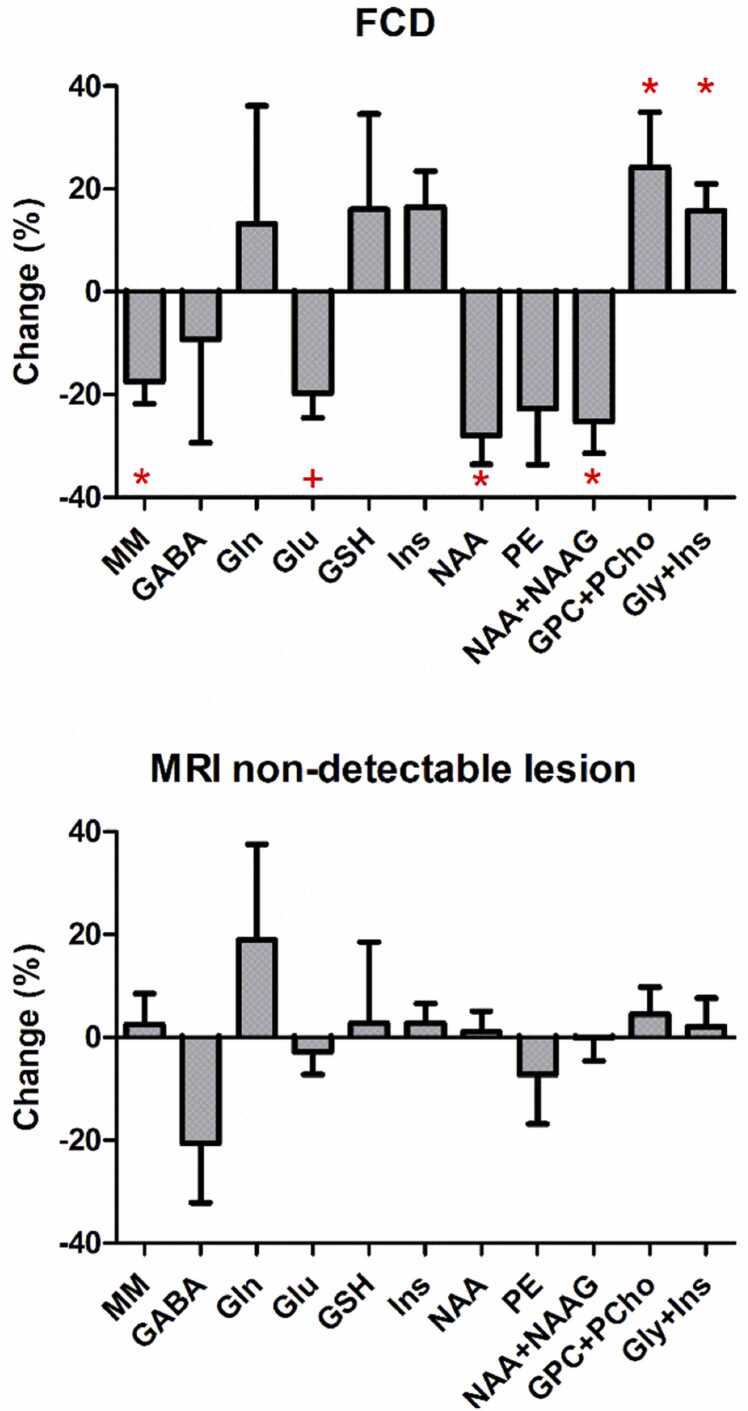
Neurochemical changes between lesion and contralateral side in epilepsy patients with the focal cortical dysplasia and MRI non-detectable lesion (* p < 0.05, + p = 0.057). The plot shows the mean percentage difference between lesion and contralateral side and error bars represent standard errors of the mean.

In comparison to the contralateral side, FCD lesions demonstrated significant reductions in macromolecule (MM, −18 %, p = 0.045) and N-acetyl aspartate (NAA, −28 %, p = 0.022), significant increases in total choline (GPC+PCho, +24 %, p = 0. 048) and glycine + myo-inositol (+16 %, p = 0.049), a distinct trend of glutamate reduction (Glu, −20 %, p = 0.057) and no changes in GABA, Gln, glutathione, myo-inositol and phosphoethanolamine levels (Fig. 2). No significant difference was found in the non-lesion patients.

## 4 Discussion

The use of 7 T spectroscopy opens new perspectives to help identify and localize with higher precision possible lesions at the origin of drug-resistant epilepsy not visible on 3 T MRI or lower fields, thanks to the improved spectral resolution and sensitivity for neurochemical profiling. Our objectives of this preliminary study were to improve detection of brain metabolites as well as to provide additional elements to identify the likely region of the lesion and to specify the region of interest for the invasive exploratory phase (the placement of intracranial electrodes).

As previously described in the studies at 3 T, there were reduced NAA, and increased total choline and myo-inositol + glycine levels in focal cortical dysplasia [7]. With the increase of spectral resolution and sensitivity available at 7 T, metabolites measurement can be largely improved especially for J-coupled metabolites like GABA and the separated measurement of glutamate and glutamine. GABA, glutamine and glutamate levels were quantified with mean CRLBs of 18 %, 12 % and 2 %, respectively. This has allowed us to assess the interplay between glutamate, glutamine and GABA in the FCD lesions.

GABA levels did not show a statistically significant difference with respect to the contralateral homotopic region. GABA is the major inhibitory neurotransmitter which was hypothesized to be decreased in an epileptogenic zone due to its role in seizure activity [19]. However, literature results, although variable, do not demonstrate clear evidence of GABA changes in epileptogenic cortex, either by microdialysis or ^1^H-MRS [20]. In the same line, our study did not show evidence of change in glutamine level [21]. Interestingly, our study shows a decrease trend of glutamate in FCD. This finding is not expected in regard of results reported in the animal model [22]. However, current in vivo results using ^1^H-MRS are mixed [20], and reduced Glu+Gln has been reported in the epileptogenic hippocampus compared with the contralateral side [23]. The reduction of Glx/tCr was also reported in the FCD patients with ^1^H edited MRSI [24]. The causes of the variation in Glu levels are multifactorial. It seems to depend on the type of epilepsy, the rate of seizure activity, the definition of the epileptogenic zone together with the voxel placement, and MRS methodologies (such as combined Glu and Gln measurement at 3 T and lower field strengths). For instance, the relatively stable glutamate concentration was observed in patients with minimal epileptic activity [25]. Furthermore, even though we do not detect morphological lesions in the suspected area, we have observed that GABA decreases, Gln increases, which appears to be a potential biomarker for non-lesional foci.

One of the main limitations of our study relates to the fact that we measured metabolite changes by MR spectroscopy in a given region (mono-voxel). With the current advance in MR spectroscopic imaging [26], [27] with a fast k-space sampling scheme covering the whole brain in a reasonable acquisition time (e.g. within 10 min), we hope to overcome this limitation in future studies and be able to improve lesion detection in these patients and thus be able to use it in daily clinical practice.

We conclude that performing MRS at ultra-high magnetic fields offers the potential to reveal metabolic changes in epilepsy lesions that may help to further understand the underlying pathophysiology of the disease.

## Funding/support

Funding was provided by the Center for Biomedical Imaging, Startup of the academic radiology department, and the Swiss National Science Foundation (grants 192749 and 209470 to SV, and 189064 to LX).

## Ethical statement

The study was approved by the ethics committee of the institution (no. 13–262).

## CRediT authorship contribution statement

**Xin Lijing:** Writing – review & editing, Writing – original draft, Validation, Methodology, Investigation, Conceptualization. **Lazeyras François:** Writing – review & editing, Resources. **Vulliemoz Serge:** Writing – review & editing, Resources. **Vargas Maria isabel:** Writing – review & editing, Writing – original draft, Validation, Supervision, Methodology, Investigation, Funding acquisition, Formal analysis, Conceptualization. **Grouiller Frederic:** Writing – review & editing, Writing – original draft, Resources. **Reymond Philippe:** Writing – review & editing, Investigation, Formal analysis. **Boto José:** Writing – review & editing, Writing – original draft, Formal analysis.

## Declaration of Competing Interest

The authors declare that they have no known competing financial interests or personal relationships that could have appeared to influence the work reported in this paper.
